# Immune activation and microenvironmental crosstalk in hairy cell leukemia

**DOI:** 10.3389/fimmu.2025.1728452

**Published:** 2026-02-05

**Authors:** Hammad Tanzeem, Eric J. Vick

**Affiliations:** 1 Department of Internal Medicine, University of Cincinnati, Cincinnati, OH, United States; 2 Division of Hematology/Oncology, University of Cincinnati, Cincinnati, OH, United States; 3 Division of Experimental Hematology and Cancer Biology, Cincinnati Children’s Hospital Medical Center, Cincinnati, OH, United States; 4 University of Cincinnati Cancer Center, Cincinnati, OH, United States

**Keywords:** BCR signaling, CXCL12–CXCR4, fibrosis, hairy cell leukemia, immune-evasion, leukemic microenvironment, MAPK pathway, TNF-α

## Abstract

Hairy cell leukemia (HCL) is an indolent leukemic B-cell malignancy that typically presents with pancytopenia and splenomegaly. Many patients achieve durable initial remissions with nucleoside analogs but ultimately relapse as leukemic cells acquire or exploit resistance mechanisms. Central to this resistance is the highly specialized leukemic microenvironment, particularly within bone marrow and splenic niches where hairy cells persist despite clearance of circulating disease. These protective niches provide CXCR4- and adhesion-dependent retention signals, cytokine support, and immune-evasion mechanisms that sustain leukemic survival, promote minimal residual disease, and ultimately drive relapse. In this Mini Review, we summarize how stromal interactions, extracellular-matrix remodeling, and disrupted immune surveillance reinforce therapeutic resistance in HCL, and how BCR and MAPK signaling interact with these circuits. Further, we highlight emerging strategies, including agents that disrupt chemotaxis, adhesion, and immune checkpoints, designed to dismantle microenvironmental support and improve the depth and durability of remission in HCL.

## Introduction

Hairy cell leukemia (HCL) is an indolent B-cell neoplasm marked by clonal proliferation of mature B lymphocytes with abundant pale cytoplasm and fine, “hairy” circumferential projections, which infiltrate the bone marrow (BM), spleen, and peripheral blood (1, 2). HCL accounts for approximately 1.4% of all leukemias, with an annual incidence of 0.3 cases per 100,000 individuals in the United States (3). The disease displays a marked male predominance (male-to-female ratio, 4:1) and a median age of 58 years at diagnosis (3). Most patients are asymptomatic at diagnosis, with pancytopenia often identified incidentally during routine assessment of blood counts (4). Cytopenias typically involve ≥ 2 hematopoietic lineages and circulating lymphoid cells are usually low. Approximately 20% of patients present with active infection, likely reflecting increased susceptibility due to neutropenia and impaired cellular immunity associated with the disease (5, 6). Opportunistic infections frequently occur later, as a consequence of CD4 T-cell depletion induced by purine nucleoside analog therapy (7). Extramedullary involvement, including lymph nodes and bone lesions, is more commonly observed in relapsed disease (8).

Diagnosis is confirmed by the identification of hairy cells in the peripheral blood and bone marrow, characteristic immunophenotypic features, and, in classic HCL, detection of the BRAF V600E mutation (2). Untreated patients with newly diagnosed HCL warrant close outpatient monitoring without treatment initiation until hematologic parameters decline and/or symptoms develop. Indications for treatment include abdominal fullness or discomfort due to splenomegaly (present in 80-85% of patients and rarely associated with splenic infarction), constitutional symptoms, recurrent infections, or significant cytopenias (absolute neutrophil count <1 × 10^9^/L, hemoglobin <11 g/dL, or platelets <100 × 10^9^/L) (2, 9, 10).

Hairy cells (HCs) are not readily categorized into a single B-cell subset, and their precise cell of origin remains uncertain. Neoplastic cells commonly express class-switched immunoglobulins and harbor somatic hypermutation in Ig variable genes, indicating prior germinal-center exposure (11). Along with a distinct promoter-methylation and gene-expression profile, these features support a phenotype most consistent with memory B cells (12, 13). Bone marrow biopsy typically demonstrates diffuse infiltration by atypical lymphoid cells with well-preserved cytoplasmic borders, producing the characteristic “fried egg” appearance (14). Ki-67 proliferation indices are very low, and silver staining reveals increased reticulin fibrosis, often contributing to a “dry tap” during attempted marrow aspiration (15). Immunophenotyping by immunohistochemistry and flow cytometry is central to confirming the diagnosis, and HCs typically display a mature B-cell phenotype with bright expression of CD19, CD20, CD22, PAX5, and CD79a, along with characteristic markers such as CD11c, CD25, CD103, CD123, and tartrate-resistant acid phosphatase (TRAP) (6, 9). CD38 expression (7–33% of cases) is associated with earlier relapse compared with CD38-negative disease (16).

Variant HCL (HCL-V) is characterized by a morphology intermediate between that of prolymphocytes and classic hairy cells, with lymphocytosis and cytopenias, prominent nucleoli, and an absence of Annexin A1, CD25, and TRAP in neoplastic cells (17). The BRAF V600E mutation is absent; instead, mutations occur downstream in MEK- and MAPK-related genes (18). Programmed death-1 (PD-1), a lymphoid receptor known to be overexpressed in several B-cell lymphomas and leukemias, appears highly expressed in classic HCL but shows low expression in HCL-V (19). The disease course of HCL-V is more aggressive, with poor responses to purine-analog therapy and shorter overall survival compared with classic HCL (9, 20–22).

**Figure 1:**
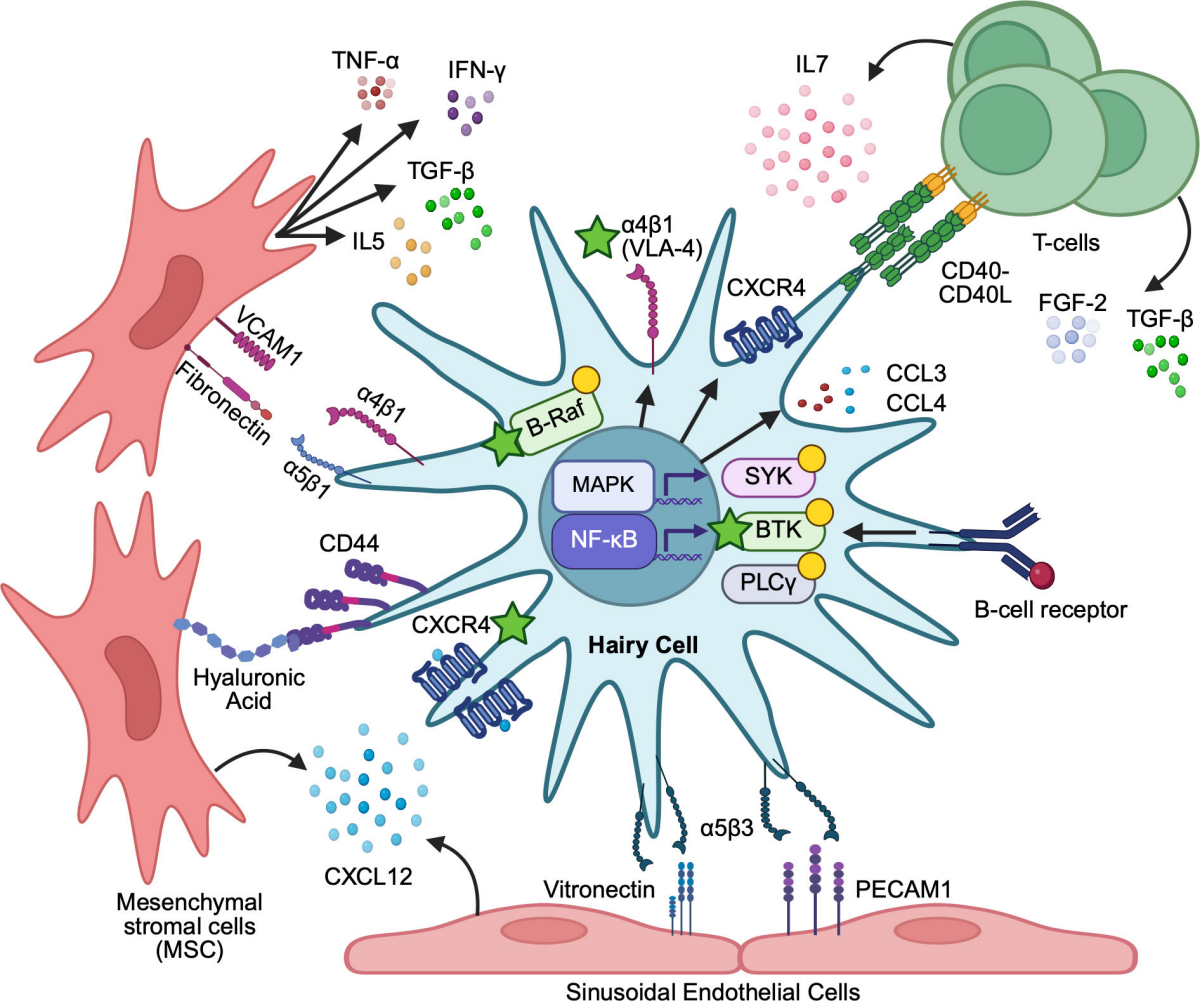
Immune activation and microenvironmental retention in classic hairy cell leukemia. Hairy cell leukemia (HCL) cells express CXCR4 and adhesion molecules (CD44, α4β1/VLA-4) that mediate binding to mesenchymal stromal cells and endothelium. Stromal and endothelial cells secrete CXCL12, promoting CXCR4-dependent migration, adhesion, and retention in bone marrow (BM) niches. Antigen-engaged B-cell receptor (BCR) signaling activates SYK, BTK, and PLCγ2 and induces CCL3/CCL4, recruiting accessory cells and T cells and reinforcing a supportive, pro-survival microenvironment. CD40–CD40L interactions and cytokines such as TGF-β and FGF-2 further augment survival cues and drive fibrosis. Together, these positive feedback loops promote immune evasion and drug resistance. Therapeutic intervention points (stars) are shown at BCR/BTK, BRAF/MEK, CXCR4, and VLA-4 nodes.

There remain significant unmet needs in the management of HCL, including greater understanding of the mechanisms sustaining minimal residual disease (MRD), and treatment options for relapse. In this Mini Review, we examine how BCR/MAPK signaling, CXCR4- and adhesion-dependent cellular retention, and immune remodeling within HCL niches cooperate to sustain leukemic cell survival, MRD, and relapse years after initial or follow-up treatment (**Figure 1**, **Table 1**). We summarize these circuits (**Table 2**) and the emerging therapeutic strategies which target them, including BTK and BRAF/MEK inhibition, agents that disrupt chemotaxis and adhesion, and emerging therapies like CAR-T cell therapy and bispecific antibodies (**Table 1**). Through these agents, likely in combination, we highlight opportunities to deepen remissions while preserving host hematopoiesis by re-wiring of the leukemic microenvironment (1, 23–25).

**Table 1:** Microenvironment-focused and targeted therapies in classic hairy cell leukemia.

Therapy/regimen	Clinical setting	Primary molecular target(s)/mechanism	Microenvironment-relevant effects	Representative outcomes	Key limitations/toxicities
Cladribine	Frontline cHCL	Purine nucleoside analog; DNA strand breaks and apoptosis in resting and dividing lymphocytes	Indirect: debulks circulating and niche-resident HCL; does not directly disrupt stromal adhesion or chemokine axes, allowing MRD in BM	ORR > 90%, CR > 75%, PFS > 20 yrs in many CRs (7)	Prolonged CD4 lymphopenia, neutropenia, infection risk; cumulative myelosuppression with repeated courses
Pentostatin	Frontline cHCL (alternative to cladribine)	Adenosine deaminase inhibitor; toxic deoxyadenosine metabolite accumulation	Similar to cladribine—effective debulking but limited direct impact on TME structure	ORR and CR comparable to cladribine; durable remissions in many patients (102)	Requires repeated dosing until max response; similar infectious and cytopenic toxicity
Purine analogue + rituximab (concurrent or sequential)	Frontline or early relapse cHCL	CD20 targeting plus purine analogue-mediated cytotoxicity	Enhances clearance of niche-associated and MRD-level disease; deeper B-cell depletion in marrow/spleen	Higher CR and MRD-negative rates than purine analogue alone; longer remission duration (88)	Infusion reactions, prolonged B-cell and CD4 depletion, infection risk
Interferon-α	Historically frontline; now niche in frail, pregnant, or heavily pretreated patients	Type I interferon; antiproliferative and pro-apoptotic signaling via JAK/STAT	Reduces IAP-1; can decrease marrow fibrosis and partially normalize hematopoiesis; modulates cytokine milieu (e.g., IL-6)	Partial responses and hematologic improvement; less commonly deep durable CR (80)	Lower response depth than purine analogues; flu-like symptoms, mood changes; slower onset of action
Obinutuzumab + vemurafenib	Frontline option for purine analogue-ineligible cHCL	BRAF V600E inhibition + type II anti-CD20	Strong debulking including niche-resident cells; may partially reverse MAPK-driven adhesion/chemokine programs	High ORR and CR rates in BRAF-mutant patients (89)	Cutaneous and systemic BRAFi toxicities; risk of secondary skin tumors; CD20-related infusion and infection risk
Vemurafenib (± rituximab/obinutuzumab)	Relapsed/refractory BRAF V600E-mutant cHCL	BRAF V600E inhibition → decreased MEK/ERK signaling	Reverses “hairy” morphology; partially overcomes stromal protection, but BMSCs can blunt ERK dephosphorylation → MRD in niches	ORR ~90–100%, CR ~35–42% in R/R HCL (103)	Short duration of response with monotherapy; cutaneous toxicity, photosensitivity, secondary skin cancers
Dabrafenib + trametinib	R/R BRAF V600E-mutant HCL, esp. after BRAFi	Dual BRAF and MEK inhibition	More complete MEK/ERK pathway shutdown; deeper reversal of adhesion/chemokine programs and niche dependence	High ORR and CR rates in small series; potential for more durable molecular remissions (104)	Pyrexia, cardiomyopathy, ocular toxicity; combination toxicity and resistance emerging
BTK inhibitors (ibrutinib, zanubrutinib)	R/R HCL; often after purine analogue and/or BRAFi; extrapolated from CLL	BTK inhibition downstream of BCR; impacts chemokine/adhesion signaling	Reduces BCR-induced CCL3/CCL4, decreases TAM/Treg recruitment; impairs CXCR4/VLA-4-mediated homing and retention	Clinical activity in small series; disease control in heavily pretreated patients (64)	Cytopenias, bleeding, atrial fibrillation (ibrutinib), infections; long-term therapy often required
Anti-CD22 immunotoxins (e.g., moxetumomab pasudotox)	R/R HCL after ≥2 prior therapies	CD22-directed toxin delivery → potent HCL-cell killing	Efficient debulking of marrow and spleen disease; may eradicate niche-resident cells if accessible	High CR rates and MRD-negative responses in many R/R patients	Capillary leak, hemolytic uremic syndrome risk; IV schedule and monitoring
Bispecific Antibodies	Investigational	CD3, CD20 bispecific	Targets minimal residual disease to prevent relapse and extend leukemia free survival.	Early trials and a case report	Cytokinereleasesyndrome,ICANS,cytopenias
Chimeric Antigen Tcell Receptor Therapy	Investigational	CD3, CD22 bispecific	Targets minimal residual disease to prevent relapse and extend leukemia free survival.	Single small trial in relapsed patients (99)	Cytokinereleasesyndrome,ICANS,cytopenias
TGF-β/fibrosis-directed agents	Investigational	TGF-β pathway inhibition; antifibrotic signaling	Potential reduction of BM fibrosis and restoration of hematopoiesis; re-shapes ECM and stromal support	Early-phase signals in solid tumors/fibrosis; not yet established in HCL	On-target toxicities; global TGF-β blockade may disrupt normal tissue homeostasis
CXCR4 antagonists (e.g., plerixafor; next-generation agents such as BPRCX807)	Investigational	Reversible CXCR4 blockade disrupting the CXCL12–CXCR4 axis on HCL cells and stromal/endothelial niches	Rapidly mobilizes BM- and splenic niche–resident HCL cells into the peripheral blood, potentially overcoming stromal protection and enhancing sensitivity to PN, BRAFi/MEKi, or BTK inhibitors; may transiently free niche space for normal hematopoiesis	No dedicated HCL trials	Transient leukocytosis, injection-site reactions, myalgias and GI upset; logistical complexity of timed combination schedules
VLA-4 blockade (natalizumab)	Investigational;Multiple sclerosis, not studied in HCL	Antibody-mediated inhibition of α4β1 integrin (VLA-4), blocking adhesion of HCL cells to VCAM-1–expressing endothelial and stromal cells	Disrupts VLA-4/VCAM-1–dependent retention of leukocytes	No dedicated HCL trials	Risk of serious infections and progressive multifocal leukoencephalopathy (PML) with sustained α4-integrin blockade

Therapeutic options for hairy cell leukemia are expanding along with the ongoing increase in targeted agents and microenvironment-focused therapies. Because of the generally high degree of response and indolent disease course, many agents have yet to be evaluated in prospective trials but may be useful in the future for improving the duration of response and approaching a functional cure. Representative outcomes are approximate ranges drawn from pivotal trials and large series cited in the main text and are not based on head-to-head comparative studies. cHCL, classic hairy cell leukemia; PN, purine nucleoside analogue; R/R, relapsed or refractory; MRD, minimal residual disease; BRAFi, BRAF inhibitor; BCR, B-cell receptor; BTK, Bruton tyrosine kinase; ECM, extracellular matrix; BTKi, BTK inhibitor; IV, intravenous; MSC, mesenchymal stromal cell; HSC, hematopoietic stem cell.

## Molecular alterations and the leukemic microenvironment

A defining molecular feature of classic HCL is the activating BRAF V600E mutation (9). Under physiologic conditions, RAS–GTP activates BRAF through conformational changes and phosphorylation within the activation segment, thereby initiating downstream MEK/ERK signaling. Upon ligand engagement at the cell surface, this cascade transduces survival and proliferative signals to the nucleus. The canonical c.1799T>A (p.V600E) substitution constitutively activates BRAF, by replacing valine with glutamate at position 600 in the kinase domain. Subsequent downstream phosphorylation of the MEK kinases (pMEK), in turn, phosphorylates the ERK kinases (pERK), sustaining nuclear ERK activity and driving transcriptional programs that support cell-cycle progression and survival (22, 24). Given the low proliferative index in HCL, sustained MAPK output is thought to primarily promote survival rather than high-level proliferation (24, 26). The BRAF V600E mutation is typically stable over time and remains detectable at relapse (27, 28). Beyond BRAF V600E, classic HCL (cHCL) cases often have recurrent co-mutations that can further alter signaling and microenvironmental programs. Inactivating CDKN1B mutations occur in around 16% of cHCL and are often clonal, consistent with an early cooperating lesion; functionally, loss of the p27 protein produced from CDKN1B may lower apoptotic thresholds and facilitate survival downstream of ERK and BCR inputs (29).

Transcriptional and chromatin regulators also contribute to HCL pathogenesis. Recurrent mutations in KLF2, a regulator of lymphocyte trafficking and adhesion programs, and in the chromatin modifier KMT2C (MLL3) have been identified in classic HCL cohorts. These alterations likely influence chemokine- and adhesion-related gene sets that govern niche retention and immune crosstalk, including CXCR4–CXCL12 and CD44–hyaluronan signaling, thereby linking genetic lesions to microenvironmental dependence (30). BRAF-wild-type HCL-V is characterized by distinct genetic aberrations. Activating mutations in MAP2K1 (MEK1) are highly prevalent in HCL-V and in BRAF-WT/IGHV cases, directly hard-wiring MEK–ERK output (18). Additional recurrent lesions in HCL-V include CCND3 and U2AF1 (each present in ~13% of discovery cohorts), which help distinguish HCL-V from classic HCL and may affect cell-cycle regulation and RNA splicing of signaling components relevant to immune activation (31).

Activation of these oncogenic signaling pathways is central to HCL pathophysiology. As previously discussed, mutations in BRAF or downstream components lead to constitutive activation of the RAF–MEK–ERK cascade. This sustained signaling contributes to the characteristic “hairy-cell” morphology, which increases surface area and facilitates interactions with microenvironmental ligands and neighboring cells, thereby enhancing contact-dependent support within bone marrow and splenic niches (22, 27). Inhibition of this pathway with BRAF-targeted therapies can reverse the hairy-cell phenotype (32). Importantly, the MAPK pathway also serves as a therapeutic target, with BRAF and MEK inhibitors demonstrating clinical efficacy, although resistance can emerge over time, in part through adaptations within the leukemic microenvironment described below (**Table 2**) (24).

## Understanding the leukemic microenvironment

Bone marrow (BM) microenvironments are complex structures that support hematopoiesis through renewal, differentiation, and expansion of cell lineages. These specialized niches are composed of cellular elements including mesenchymal bone marrow stromal cells (BMSC), endothelial, immune, and accessory cells, osteoblasts and osteoclasts as well as the extracellular matrix (ECM) (33). BMSCs in these specialized niches express ligands for chemokine receptors and adhesion molecules, which are critical for migration, adhesion, and retention of progenitor cells in the BM (23). Hairy cells utilize these mechanisms to gain access to and occupy these protective niches, promoting HC survival and limited proliferation as well as contributing to eventual therapy resistance (23, 34).

## Chemokine and cytokine networks remodel the leukemic microenvironment

The chemokine receptor CXCR4 and its ligand, CXCL12, are important components of immune crosstalk between hairy cells and the leukemic microenvironment, and overexpression of CXCR4 is linked to disease progression, leukemic cell survival, and chemoresistance (**Figure 1**) (35). Within the bone marrow, sinusoidal endothelial cells express CXCL12, which drives migration of HSCs from the quiescent endosteum to vascular niches where they can multiply, establishing a supply of mature cells that are released into the bloodstream (36, 37). In the spleen, red-pulp expansion with blood-filled pseudosinuses and white-pulp atrophy are characteristic; hairy cells line vascular spaces and surround erythrocyte clusters, reflecting niche remodeling analogous to that seen in the marrow (14). A relative deficiency of CXCR5/CCR7 and strong CXCR4 expression favor BM, hepatic, and splenic homing/retention over lymph-node trafficking, thereby biasing HCs toward protective extranodal niches (38). Within hepatic and splenic niches, HC release TNF-α, which induces VCAM-1 on endothelium and reinforces α4β1 (VLA-4)-dependent adhesion, further stabilizing niche occupancy (**Figure 1**). Histologically, splenic pseudosinuses and hepatic vasculature classically reflect this niche remodeling (39–41). T-cells in these niches secrete cytokines including IL-3 and GM-CSF, necessary for hematopoiesis and maintenance of stem cells. They not only provide protection from autoreactive immune cells, but also contribute to a cytokine milieu that can be co-opted by HCs (42, 43).

Expression of these chemokines drives tissue homing and leukemic microenvironment remodeling and also mediates treatment response to therapy and innate immune evasion. These cytokines interact with the leukemic microenvironment to induce inhibitor of apoptosis protein-1 (IAP-1) in leukemic cells, thereby mediating resistance to chemotherapy, explaining why circulating HCs are reduced during therapy, while those in the bone marrow survive (44). Moreover, in other cell models such as T-cells and endothelial cells, integrins lead to activation of NF-κB signaling, which mediates cell survival (45, 46). A recent study using genomic and transcriptional data of HCL cell lines and patient material further underlined the importance of NF-κB signaling in hairy cells, supporting a central role for this pathway in microenvironment-driven survival (47).

### Bone marrow fibrosis results from ECM remodeling

Myelosuppression in HCL is driven in part by a reduction of the circulating progenitor cell compartment and direct suppression of hematopoiesis. These effects can be independent of both the degree of marrow fibrosis and the extent of infiltration by malignant cells (48). TNF-α secreted by leukemia cells directly contributes to the inhibition of progenitor cells in the bone marrow and, when coupled with a relative paucity of colony stimulating factors needed for HPC differentiation into myeloid progenitor cells, contributes to hematopoietic dysfunction in HCL (49). For example, monocytopenia is nearly universal in classic HCL and likely reflects both disease biology and niche remodeling, although precise mechanisms remain incompletely defined (1, 23). Deficient production of hematopoietic growth factors by accessory cells may drive pancytopenia, a hypothesis supported by the ability of G-CSF to restore progenitor maturation and elevate peripheral blood counts (49).

Bone marrow fibrosis is driven by secretion of fibrogenic cytokines such as fibroblast growth factor (FGF-2) and transforming growth factor β (TGF-β), which trigger the production and subsequent organization of a fibronectin matrix by neoplastic cells, contributing to niche remodeling and hematopoietic failure (**Table 2**). Without targeted immunohistochemistry, this markedly hypocellular marrow can occasionally mimic aplastic anemia (50). CD44 on HCs interacts with hyaluronan in the BM and promotes autocrine secretion of FGF-2. FGF-2 is not only secreted by leukemic cells, but these cells express FGFR1, which, when activated by FGF-2, drives cell cycle progression and the production and secretion of fibronectin (51–55). In the absence of hyaluronan, TGF-β produced by HCs sustains fibrosis by inducing adjacent bone marrow fibroblasts to synthesize and deposit extracellular proteins. These fibroblasts predominantly secrete type III collagen, which forms the fine reticulin meshwork characteristic of HCL (56). TGF-β exerts a dose-dependent effect on marrow fibroblasts, and elevated circulating levels can further intensify bone marrow fibrosis (57). HCs also enhance cytokine-mediated adhesion to fibronectin through overexpression of FLT3 and IL-3Rα, which activate the physiologic fibronectin receptors VLA-4 and VLA-5 (45). Finally, overexpression of metalloproteinase inhibitors such as RECK, TIMP1, and TIMP2 by HCs reduces ECM degradation, in part by increasing collagen fiber stability, thereby further promoting bone marrow fibrosis (12, 46).

Targeted therapies to limit and reverse BM fibrosis could restore normal hematopoietic function and offer durable symptom relief, highlighting fibrosis as a modifiable microenvironmental component. One promising therapeutic target is TGF-β, which drives marrow fibrosis and subsequent pancytopenia through progressive accumulation of collagen and extracellular matrix (**Table 2**). Preclinical studies have shown the efficacy of TGF-β-directed pharmacologic agents through reductions in circulating in circulating TGF-β levels and decreased transcriptional activity of downstream genes (47, 58). Despite this promise, clinical application remains limited by disruption of normal physiological pathways and the systemic toxicities associated with TGF-β inhibition (59). A more comprehensive understanding of TGF-β and FGFR signaling in HCL may enable the development of combination therapies that more precisely modulate the leukemic microenvironment while preserving normal hematopoietic niche function.

**Table 2:** Key cytokine and growth-factor effectors shaping the hairy cell leukemia microenvironment.

Chemokine / Cytokine / Axis	Primary Source(s)	Receptor / Target	Main Biological Function(s)	Immunosuppressive/Pro-survival Pathways
**CXCR4–CXCL12**	Endothelium; HCs	CXCR4	Retention in BM/splenic niches; Reduced trafficking to lymph-node	Protection from chemotherapy; Microenvironment-mediated apoptosis resistance (increased IAP-1)
**CCL3/CCL4**	HCs after BCR engagement	CCR1/CCR5 on T cells, monocytes, macrophages	Recruitment of Reg T cells; Recruitment of tumor-associated macrophages (TAMs)	TAM-mediated immune suppression; Amplification of pro-survival and pro-fibrotic signaling; Suppressed by BTK inhibitors (e.g., ibrutinib)
**TNF-α**	HCs	TNF receptors on endothelial cells, progenitors	Induces VCAM-1 on endothelium; Enhances VLA-4-dependent adhesion;Suppresses hematopoietic progenitors	Stabilizes leukemic niches; Contributes to myelosuppression;Supports chronic inflammatory yet immunosuppressive milieu
**IL-3/GM-CSF**	T cells in leukemic niches	IL-3R, GM-CSFR on progenitors and HCs	Support hematopoiesis and stem cell maintenance; Enhance adhesion to fibronectin	Co-opted to support leukemic survival; Reinforces niche dependence and persistence
**IL-10**	HCs	IL-10R on T cells	Anti-inflammatory cytokine	Direct suppression of effector T-cell activation and proliferation;Promotes immune tolerance
**TGF-β**	HCs	TGF-β receptors on fibroblasts, immune cells	Induces marrow fibrosis; ECM deposition and fibroblast activation	Suppresses immune responses; Promotes immune exclusion and hematopoietic failure
**FGF-2**	HCs (autocrine loop) via CD44–hyaluronan	FGFR1 on HCs and fibroblasts	Fibronectin production; Cell-cycle progression	Enhances niche remodeling; Indirect immune suppression via fibrosis
**Soluble IL-2 receptor**	HCs	Sequesters IL-2	Acts as IL-2 “sink”	Inhibits cytotoxic T-cell and NK-cell expansion; Favors Treg development and immune tolerance

Selected chemokines, cytokines, and growth factors implicated in leukemic niche retention, immune evasion, and marrow fibrosis in HCL are shown, with their principal cellular sources and functional effects on leukemic cells and the surrounding microenvironment. BM, bone marrow; ECM, extracellular matrix; HC, hairy cells; MSC, mesenchymal stromal cell; HSC, hematopoietic stem cell; MMP, matrix metalloproteinase; IL, interleukin; TNF, tumor necrosis factor; TGF, transforming growth factor.

## BCR signaling and T-cell–mediated immune dysregulation in HCL

Engagement of the B-cell receptor (BCR) is a key event in the pathogenesis of HCL and has therefore become an important target for novel therapeutic strategies, particularly those aimed at disrupting microenvironmental signaling. Bruton tyrosine kinase (BTK) is a central mediator of BCR signaling and contributes to chemokine-receptor and adhesion-molecule signaling in both normal and malignant B cells (60). Upon BCR stimulation, HCs secrete the chemokines CCL3 and CCL4, which in turn recruit regulatory T cells and tumor-associated macrophages (TAMs). TAMs promote carcinogenesis by acting on accessory cells within the leukemic microenvironment, including fibroblasts and endothelial cells, thereby amplifying pro-survival and pro-fibrotic cues (61). As an example, ibrutinib ablates BCR-dependent CCL3/CCL4 induction in B-cell malignancies and suppresses survival/trafficking signals relevant to HCL biology (62). BCR signaling also triggers downstream phosphorylation of BTK, ERK, and AKT. Both ibrutinib and second BTK inhibitors abrogate these signaling pathways, reducing HC survival (63, 64).

Within these microenvironments, BMSCs interact with VLA-4 expressed on neoplastic cells, triggering both NF-κB downstream pathways and MAPK pathways, which stimulate prolonged survival and limited proliferation of malignant cells (25). A majority of HCL patients have leukemic cells characterized by mutated immunoglobulin variable region genes (M-IGHV) (65). Unmutated forms (UM-IGHV) found in a minority of cases are more responsive to BCR triggers and typically have rapid progression of disease, when compared to their mutated counterparts (66). BCR cross-linking by antigen activates both kinases and signal transducers, through a phosphorylation cascade. These include spleen tyrosine kinase (SYK), Bruton’s tyrosine kinase (BTK), and phospholipase C gamma (PLCγ2). Downstream intracellular calcium mobilization leads to activation of NF-κB and MAPK that support carcinogenesis (67). BCR engagement also triggers the release of the chemokines CCL3 and CCL4 by normal and malignant B-cells, and these coordinate monocytes and T-cell recruitment to the tissue sites where activated B-cells reside (**Figure 1**) (39). Together, all these steps promote B-cell proliferation and confer a survival advantage, particularly within sanctuary niches of the tumor microenvironment.

In HCL, T-lymphocyte function is impaired, as T-cells exhibit clonal expansion and a skewed repertoire of the T-cell receptor β variable region (68). Such T-cells recognize and undergo subsequent activation by CD40-activated HCL, which leads to leukemia progression instead of disease suppression (69). CD40–CD40L engagement amplifies survival signaling and augments BCR-induced CCL3/CCL4 production, reinforcing accessory-cell recruitment (23, 70). In addition to direct T-cell suppression, antigen presentation is impaired through a paucity of dendritic cells, which are essential for adaptive immune response (71). While likely related to decreased marrow monocyte counts, further effects on dendritic cell populations have not been fully evaluated. T-cell function is further impaired by HC secretion of inhibitory cytokines like IL-10, which dampen the activation and proliferation of effector T-cells (70, 72).

Hairy cells downregulate expression of CD11a and CD54 which are critical for enhancing B-cell proliferation and differentiation, as well as for the production of IL-2 by activated CD4+ T-cells (73). IL-2 is thought to control differentiation and homeostasis of both pro- and anti-inflammatory T-cells (74). Primarily produced by activated T cells, it acts as an autocrine growth factor for T cells and a paracrine growth factor for NK cells. At high levels, IL-2 activates and supports expansion of the T-cell compartment, especially cytotoxic T-cells, while low levels support T-regulatory cell development, promoting immune tolerance (75). The soluble IL-2 receptor, produced and released by HCs, forms a sink for IL-2 in the microenvironment, thereby inhibiting T-cell activation and fostering an anti-inflammatory, tolerance-promoting environment (74). Thus, malignant cells may evade immune responses by lowering IL-2–mediated immune activation through increased secretion of the soluble IL-2 receptor, contributing to a chronically immunosuppressed microenvironment (76).

Finally, in a study of the bone marrow microenvironment by Koldej et al, HCL was associated with the dysregulation and decreased expression of multiple immune checkpoints including CTLA-4, VISTA, and STING (77). MHC-Class II expression is increased on HCs cells in patient samples prior to treatment and does not return to the levels of healthy controls even after treatment. Treatment response was associated with an increase in the expression of CD3 and CD8 compared to non-responders, suggesting partial restoration of cytotoxic T-cell surveillance within the marrow niche (77). Together, these findings highlight broad immune-checkpoint dysregulation and a partially reversible impairment of antitumor T-cell immunity in HCL.

## Current strategies to target the leukemic microenvironment in hairy cell leukemia

Protective spaces in the bone marrow are instrumental in early development and proliferation of leukemia cells, but within these niches HCs can remain resistant, evading standard pharmacotherapy that eliminates the bulk of cancer cells (22). Residual leukemia cells lurk in these protective microenvironments where they continue to receive signals promoting survival and limited growth. Unsurprisingly, the bone marrow is a well-documented locus of minimal residual disease and the primary source of relapse in patients with HCL (78).

Interferon-α was among the earliest therapies available and exerted its cytotoxic effect on leukemia cells by stimulating autocrine production of TNF-α, which led to suppression of inhibitor of apoptosis protein-1 (IAP-1) expression and accumulated intracellular Ca^2+^ ions, thus curtailing neoplastic cell survival (44, 79, 80). Interferon-α’s therapeutic effects also extended to a reduction in bone marrow fibrosis and subsequent improvement of marrow-fibrosis associated hematopoietic dysfunction (81). One explanation of interferon-α-induced hematopoietic regeneration is through increased secretion of IL-6 by leukemia cells when treated with interferon-α *in vitro*. Leukemic-cell interactions with vitronectin and fibronectin in the ECM of the BM prevent this interferon-mediated suppression of IAP-1, outlining yet another example of how leukemic cells are protected in these niches (**Figure 1**) (54).

Treatment for HCL is indicated if patients have symptoms from the disease or if their hematologic parameters are declining. First-line treatment remains conventional chemotherapy with the purine analogues, cladribine or pentostatin. Treatment has similar outcomes, although cladribine is often preferred as side effects may be better tolerated. In addition, single-cycle administration of cladribine over 5–7 days makes for easier delivery compared to pentostatin, which is typically administered every other week and continued until maximal response, often over several months (82, 83). These drugs have an overall response rate (ORR) > 90%, CR > 75%, and long-term progression-free survival (PFS) longer than 20 years for people in complete remission (CR) (84, 85). Side effects are similar and include T-cell lymphopenia (particularly a long-lasting depletion of CD4+ cells), neutropenia, rash, and increased susceptibility to infections (86). Combination of cladribine with anti-CD20 monoclonal antibodies like rituximab either concurrently or sequentially, leads to remission in most HCL patients. Concomitant or sequential rituximab with pentostatin deepens responses and more frequently achieves undetectable minimal residual disease (MRD), translating into longer remission durations compared to pentostatin alone (87, 88). In HCL-V, adding rituximab is strongly recommended because of the inherent chemoresistance to purine analogues (88). For patients who are unable to tolerate or receive purine analogs, the combination of obinutuzumab and the BRAF inhibitor vemurafenib has a comparably high remission rate in front-line treatment and may offer an alternative route to deep remissions via simultaneous targeting of BCR, MAPK and the leukemic microenvironment (89). A summary of key therapeutic regimens, their primary molecular targets, microenvironment-relevant effects, and representative outcomes is provided in **Table 1**.

Although treatment with purine analogues achieves durable remission in most HCL patients, up to 25% present with relapsed and/or refractory disease within 5 years (1). Such patients are difficult to treat, become progressively less sensitive to purine analogues, have a shorter median duration of response after successive relapses, and are at higher risk of having a shorter overall survival, emphasizing the contribution of microenvironmentally protected residual disease (5, 90). Therapeutic regimens for refractory or relapsed HCL depend on duration of first response and the agents used to date. Patients who remain in CR for more than five years may be retreated with the initial first-line therapy. In cases where remission lasted between 2–5 years, retreatment with purine analogues combined with rituximab should be considered. Patients who relapse within 2 years, are refractory to chemotherapy, or have relapsed multiple times, are all candidates for BRAF inhibitors which can be combined with rituximab or obinutuzumab. Vemurafenib, dabrafenib, and encorafenib can be considered in the approximately 85% of patients with BRAF V600E mutations (82). Vemurafenib demonstrates an ORR of 90-100%, including a 35-42% CR rate in relapsed or refractory HCL (91). Though it has a high response rate, monotherapy usually has a short duration of response, consistent with persistence of microenvironmentally protected clones. Mechanistic work indicates that BMSCs blunt BRAF-MEK-ERK dephosphorylation and pro-apoptotic output, consistent with VLA-4/CXCR4-mediated stromal protection (32, 34). Rational combinations that include microenvironmental disruptors could improve both the depth and durability of responses.

Further progression beyond BRAF inhibitors has led to co-inhibition of BRAF and MEK with dabrafenib and trametinib (9). The latter combination causes strong MEK/ERK dephosphorylation and consequently decreases transcriptional output of the pathologically activated BRAF-MEK-ERK pathway. This effect is manifested by loss of the “hairy cell” morphology and apoptosis of these neoplastic cells and has the added advantage of bypassing resistance to BRAF inhibitor monotherapy, which is often mediated by reactivation of alternative signaling within the MEK/ERK pathway (32, 92). Clearly, cells present in the HCL leukemic microenvironment have the capacity to circumvent changes induced by certain drugs, not only fostering leukemic progression but also profoundly undermining the efficacy of conventional and emerging cancer therapeutics.

## Future directions to address resistance in hairy cell leukemia

Treatment of relapsed or refractory disease continues to be a challenge even with novel targeted therapies, underscoring the need for a deeper understanding of the molecular signaling that shapes interactions between neoplastic cells and their microenvironment. The intricate crosstalk among diverse immune-cell populations and their engagement with both cellular and acellular components of this protective niche remain incompletely understood. Adding to this complexity is substantial heterogeneity within the microenvironment itself - not only across different HCL variants but also among individual patients. These complexities make it difficult to identify broadly applicable targets for interventions that disrupt the leukemic microenvironment, while still preserving physiologic architecture and tissue homeostasis.

Even among patients achieving a CR, MRD may persist in the blood and bone marrow, highlighting the limited curative potential of existing therapies. While the clinical utility of MRD monitoring in CR remains debated and is not recommended for routine practice, accumulating evidence suggests that MRD negativity is associated with longer treatment-free survival (1, 93). With successive retreatment using purine analogues, progression-free survival declines while toxicities, including myelotoxicity and secondary malignancies increase (94). Consequently, there is an ongoing need for therapies that more effectively eradicate MRD while maintaining acceptable tolerability. Newer immunotherapeutics that have been approved by the FDA to treat other B-cell malignancies include CD3/CD20 bispecific antibodies (epcoritamab, mosunetuzumab and glofitamab) developed for diffuse large B-cell and follicular lymphomas and which have demonstrated impressive activity as monotherapies in patients with heavily pre-treated, relapsed, or refractory disease (95). A single case was published, using the bispecific mosunetuzumab in a heavily pre-treated patient which resulted in CR (96). These bispecific antibodies cause predictable immune toxicities, chiefly cytokine release syndrome, low-grade immune effector cell-associated neurotoxicity syndrome, as well as cytopenias and infection risk from sustained B-cell depletion and T-cell activation. Risk mitigation requires step-up dosing, steroid prophylaxis, IL-6 blockade availability, and close early-cycle monitoring independent of molecular design or route. Although feasible in the outpatient setting, clinical use remains operationally complex, and treatment duration (fixed vs continuous) significantly influences cumulative toxicity and risk of infection risk (97). Moxetumomab pasudotox established that CD22 is a highly effective therapeutic target in HCL, reflecting near universal expression of CD22 in both classic HCL and HCL variant (HCLv). Since CD22 density is much higher in HCL compared with B-ALL, CD22-directed cellular therapies represent a promising option for patients with relapsed/refractory disease on purine analogues and BRAF-directed regimens (98). A phase 1 trial of a CD22-directed CAR T-cell therapy that include patients with HCL is currently ongoing (NCT04815356). As also observed with B-ALL, higher disease burden at the time of CAR T-cell infusion among early participants was associated with increased toxicities, supporting tumor debulking prior to CAR T-cell infusion as a strategy to mitigate toxicity as enrollment continues (99). The effects of therapy-induced cytokine release represent a unique mechanism of possible resistance to bispecific and CAR T-cell therapy, which has also been noted in attempts to design CAR-T cells for the treatment of acute myeloid leukemia. Myeloid-supporting cytokines secreted during cell therapy promote leukemic survival through kinase signaling, leading to CAR T-cell exhaustion (100). This cytokine-mediated, therapy-induced resistance mechanism is distinct from those described in B-cell malignancies and underscores how dissecting tumor microenvironment driven resistance mechanisms can provide a framework for understanding and addressing therapeutic resistance in HCL and beyond.Additional avenues include CXCR4 antagonists (e.g., plerixafor, BPRCX807) and VLA-4 blockade to transiently mobilize HCs from protective niches, possibly improving the efficacy of BCR/MAPK-directed agents (34). Chief among these would be CXCR4 antagonists (plerixafor, BPRCX807), which would antagonize a major signaling hub in HCL. VLA-4 also represents a novel therapeutic target, already used in multiple sclerosis and may be leveraged to prevent HCL niche occupation and growth (101). Combination regimens such as those extrapolated from CLL, pairing a BTK inhibitor with an anti-CD20 antibody, or integrating a BRAF inhibitor in refractory disease, also warrant exploration. Although toxicity concerns have historically limited their use, combinations may become viable options with careful attention to sequencing, concurrent administration strategies, and infection-risk monitoring. Taken together, current data suggest that future progress in HCL will depend on integrating tumor-intrinsic targeting (BRAF/MAPK, BTK) with microenvironment-directed strategies that modulate chemokine axes, adhesion pathways, and fibrogenic signaling.

Rational avenues include CXCR4 antagonists (e.g., plerixafor, BPRCX807) and VLA-4 blockade to transiently mobilize HCL cells from protective niches, possibly improving the efficacy of BCR/MAPK-directed agents (34). Chief among these would be CXCR4 antagonists (plerixafor, BPRCX807), which would antagonize a major signaling hub in HCL. VLA-4 also represents a novel therapeutic target, already used in multiple sclerosis and may be leveraged to prevent HCL niche occupation and growth (92). Combination regimens such as those extrapolated from CLL, pairing a BTK inhibitor with an anti-CD20 antibody, or integrating a BRAF inhibitor in refractory disease, also warrant exploration. For patients who have exhausted purine analogues and BRAF-directed regimens, CD22-directed recombinant immunotoxins such as moxetumomab pasudotox provide an additional option to debulk CD22-high disease in marrow and spleen, with high complete remission and MRD-negative rates in heavily pretreated cohorts, albeit at the cost of infusion-related toxicity and careful monitoring requirements (93). Although toxicity concerns have historically limited their use, such combinations may become viable options with careful attention to sequencing, concurrent administration strategies, and infection-risk monitoring. Taken together, current data suggest that future progress in HCL will depend on integrating tumor-intrinsic targeting (BRAF/MAPK, BTK) with microenvironment-directed strategies that modulate chemokine axes, adhesion pathways, and fibrogenic signaling.
